# Case Report: From diagnosis to therapy: a lung ultrasound-driven precision strategy for neonatal atelectasis management

**DOI:** 10.3389/fped.2025.1584262

**Published:** 2025-06-05

**Authors:** Qi Chen, Wei Xiong, Li Jun

**Affiliations:** Neonatal Intensive Care Unit, Shangrao Children’s Hospital, Shangrao, Jiangxi Province, China

**Keywords:** neonatal atelectasis, lung ultrasound, pneumonia, meconium aspiration syndrome (MAS), respiratory distress syndrome (RDS)

## Abstract

**Objective:**

To examine the clinical value of lung ultrasound (LUS) in the individualized management of neonatal atelectasis and assess its effectiveness in directing condition-specific treatment strategies.

**Methods:**

Three neonatal atelectasis cases with differing causes, pneumonia, pulmonary hemorrhage, and meconium aspiration syndrome, were treated with LUS-guided, personalized interventions. These included ultrasound-directed airway clearance, selective bronchoalveolar lavage, and high-frequency chest wall oscillation.

**Results:**

LUS enabled continuous, real-time assessment of atelectasis severity and distinct pathological signs such as hepatization and the shred sign. This imaging guidance allowed targeted therapies that shortened hospitalization by an average of 40%. In all cases, lung re-expansion was achieved without adverse events.

**Conclusion:**

Due to its lack of radiation, high sensitivity, and real-time feedback, LUS offers a valuable tool for guiding individualized, etiology-specific therapies in neonatal atelectasis. It presents a clinically adaptable approach for optimizing outcomes in this population.

## 1 Foreword

Neonatal atelectasis, often arising from severe respiratory illness, continues to be difficult to diagnose and manage effectively. Traditional imaging modalities expose newborns to repeated radiation, raising long-term safety concerns (1). Neonatal lung ultrasound (LUS), a rapidly advancing imaging method, offers high diagnostic sensitivity, real-time visualization, and eliminates radiation exposure, outperforming both x-ray and CT in many contexts (2). In addition to its diagnostic strengths, LUS also plays a guiding role in therapy. This paper explores its application in treating three representative causes of neonatal atelectasis, neonatal COVID-19 (3), pulmonary hemorrhage (4), and MAS (5), with ultrasound-directed interventions.

## 2 Standardized ultrasound equipment specifications

All cases were assessed using the Mindray M9 Super ultrasound system with a 12–4 MHz transducer. The device was manufactured by Shenzhen Mindray Bio-Medical Electronics Co., Ltd., located at Mindray Building, Keji 12th Road South, Nanshan High-Tech Industrial Park, Shenzhen, Guangdong Province, China.

## 3 Case description

### 3.1 Case 1

A 14-day-old male infant, delivered by cesarean section at 38 weeks and 5 days of gestation, presented with fever (38°C–39°C) and dyspnea. Admitted to hospital on February 18, 2024. The mother was confirmed COVID-19 positive at the time of delivery. The infant had developed a fever two days prior, with body temperatures ranging between 38°C and 39°C, and gradually worsening shortness of breath. The family sought medical care at our hospital after symptoms intensified.

On physical examination, the infant had a temperature of 38.5°C, respiratory rate of 62 breaths/min, heart rate of 180 beats/min, weight of 4.0 kg, and oxygen saturation of 87%. Signs of increased respiratory effort were evident, with three notable retraction signs, bilaterally symmetrical breath sounds, and audible sputum in the airway. No abnormalities were found in the cardiovascular, abdominal, or neurological systems.

#### 3.1.1 Imaging studies

**Figure 1:**
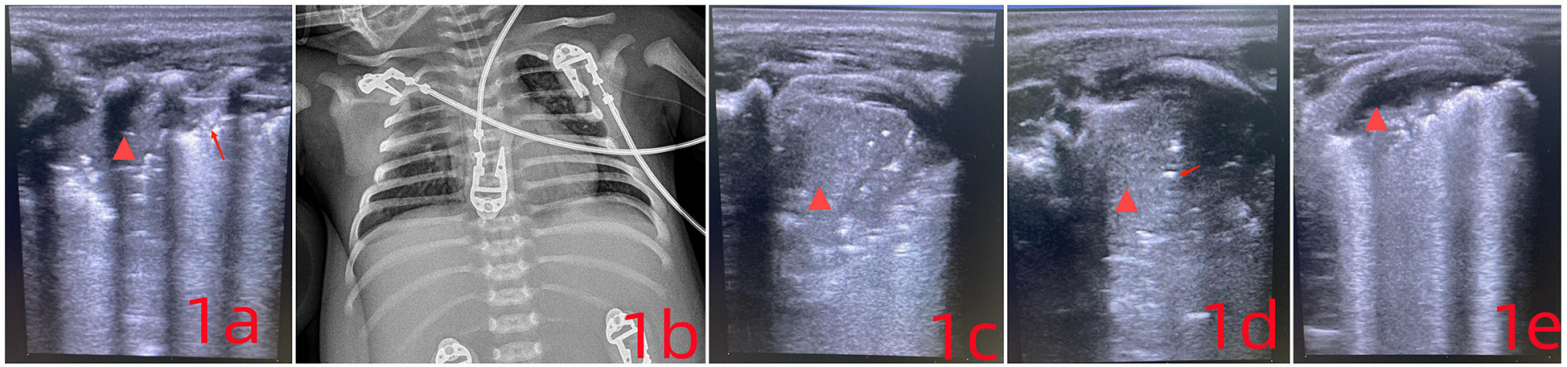
**(a)** the arrow points to the disappearance of the pleural line, while the 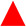 symbol marks a region of severe pulmonary consolidation. The lung tissue appears hepatized, showing liver-like hypoechogenicity, with an absent pleural line and visible air bronchograms. **(c)** Compared to **(a)**, the area of hepatization has increased. The absence of air bronchograms in the lung parenchyma now indicates atelectasis. **(d)** After sputum clearance, follow-up imaging shows the reappearance of air bronchograms (indicated by arrows), signaling improvement in the previously collapsed region. **(e)** The extent of atelectasis has decreased significantly.

Lung ultrasound (LUS) showed bilateral B-lines and a consolidation area with atelectasis in the right posterior lung field (R5), extending across 2–3 intercostal spaces (Figure 1a). On LUS, pneumonia is indicated by subpleural consolidation, while atelectasis appears as a liver-like echotexture (hepatization), with the absence of dynamic bronchial inflation signs. Scanning was performed in six lung zones: anterior (sternal border to anterior axillary line) (L1–2; R1–2), lateral (anterior to posterior axillary line) (L3–4; R3–4), and posterior (posterior axillary line to paravertebral region) (L5–6; R5–6) (6).

A chest x-ray confirmed neonatal pneumonia (Figure 1b). COVID-19 nucleic acid was detected in throat swab specimens, and a CT scan revealed atelectasis in the upper right lung along with signs of neonatal pneumonia.

#### 3.1.2 Key LUS features of pneumonia include

(1) Abnormal pleural line (e.g., disappearance, irregularity, discontinuity, or blurring). (2) Lung consolidation (subpleural regions showing liver-like echotexture with bronchial inflation signs) Additional findings may include absence of pleural sliding and presence of pleural effusion. Among these, dynamic bronchial inflation and subpleural consolidation are the most informative. In severe infections, fragmented signs and hepatization are frequently observed.

#### 3.1.3 Treatment

COVID-19 infection was confirmed through nucleic acid testing. The infant was treated with mechanical ventilation, LUS-guided airway clearance, and anti-inflammatory therapy. Upon admission, comprehensive assessments of pulmonary lesions were carried out using auscultation, lung ultrasound (LUS), x-ray, and computed tomography (CT). The child was managed with invasive conventional mechanical ventilation and proactive airway clearance, maintaining a blood oxygen saturation of 95%.

After 24 hours, the infant again showed signs of dyspnea, along with patient-ventilator asynchrony and a drop in blood oxygen levels. LUS revealed worsening atelectasis in the R5 region, with hepatization and visible lung pulsation. The collapsed area had enlarged, extending across 4–5 intercostal spaces (Figure 1c).

Due to significant atelectasis in the right upper lobe, the infant was positioned prone to support postural drainage. Under real-time LUS guidance, high-frequency chest wall oscillation (HFCWO) was carried out, targeting the consolidated segment in the right upper lobe. The method involved manual vibratory percussion using a cupped-hand technique, fingers slightly flexed to distribute force evenly across the affected region. Each session lasted no more than 10 minutes and could be repeated based on clinical need. LUS reassessment followed each session to monitor treatment effectiveness (Figure 1d). Focused ultrasound monitoring was continued in areas where drainage remained inadequate. Fentanyl was administered at 1 μg/kg/h for analgesia. Considering the clinical course and potential excessive inflammatory response, intravenous immunoglobulin was given at 1 g/kg. On the next day, low-dose dexamethasone at 0.25 mg/kg was initiated every twelve hours (7–9).

With targeted respiratory therapy, ventilatory support, and pharmacologic treatment, the infant's lung condition steadily improved (Figure 1e). By day 3, the tracheal tube was removed and replaced with nasal continuous positive airway pressure (NCPAP), and the infant was discharged one week later (Discharged from the hospital on February 25, 2024). Follow-up after discharge showed no recurrence, with normal feeding, stable breathing, and appropriate weight gain.

### 3.2 Case 2

A 5-day-old preterm female infant, born at 29 weeks and 3 days of gestation with a birth weight of 700 g, was admitted due to respiratory distress. Admitted to hospital on May 10, 2024. An emergency cesarean section had been performed because of twin transfusion syndrome and oligohydramnios. The infant experienced birth asphyxia at delivery, was successfully resuscitated, and transferred to the NICU for further management.

**Figure 2:**
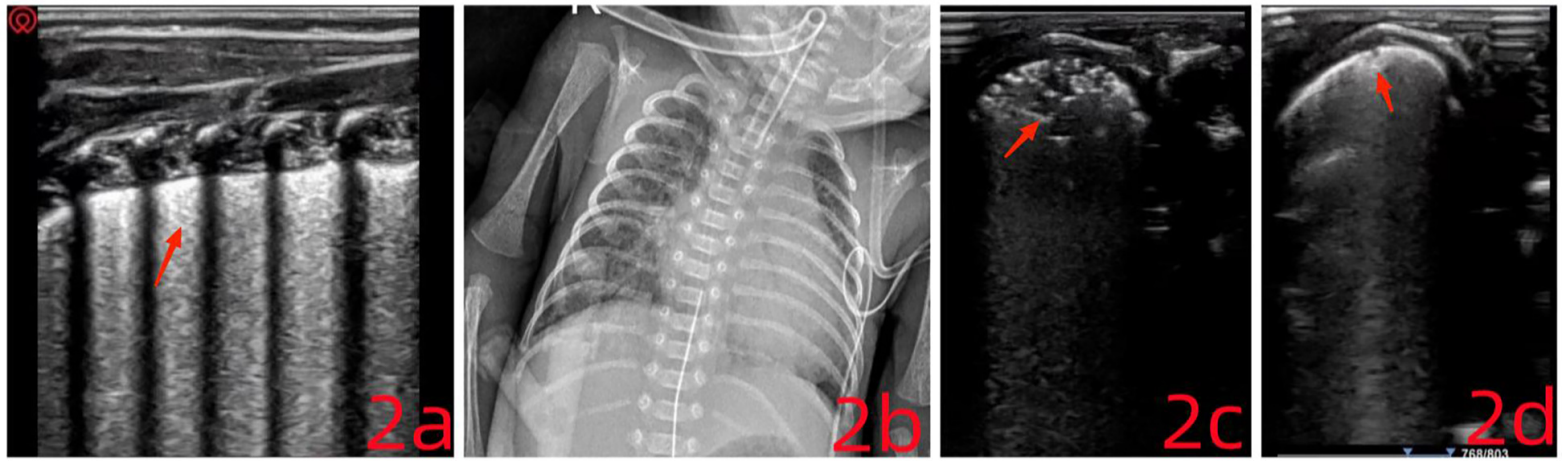
**(a)** the pleural line is no longer visible. Minor consolidation is present near the pleural surface, displaying a characteristic “snowflake sign.” **(c)** The consolidation area has expanded and appears irregular, presenting a “fragmentation sign.” **(d)** The fragmentation sign has resolved, leaving only a small subpleural consolidation.

On physical examination, the infant had a body temperature of 35°C, pulse rate of 167 beats/min, respiratory rate of 64 breaths/min, and blood pressure of 48/32 mmHg. She appeared premature, with weak responsiveness, dyspnea, chest retractions, and reduced breath sounds accompanied by scattered crackles. Cardiac auscultation revealed a regular rhythm with strong heart sounds and no murmurs over any valve area. Laboratory findings: Arterial blood gas analysis indicated a pH of 7.33, PCO_2_ of 50 mmHg, PO_2_ of 85 mmHg, lactate concentration of 8.2 mmol/L, BE of 0 mmol/L, and HCO_3_^−^ of 26.4 mmol/L. LUS: Imaging revealed scattered B-lines, disappearance of A-lines, and loss of pleural lines in the L6–R6 regions. Dynamic air bronchograms were observed along with the snowflake sign (Figure 2a). These abnormalities extended across 2–3 intercostal spaces and involved two lung zones, consistent with ultrasound features of neonatal respiratory distress syndrome.

#### 3.2.1 Main treatment course

The infant was treated with invasive mechanical ventilation and pulmonary surfactant, maintaining oxygen saturation above 95%. Gradual parameter reduction was observed during the course of ventilation. At 4 hours post-intervention, follow-up LUS showed that the snowflake sign had disappeared in both dorsal lung fields. On the following day, the ventilator was discontinued, and non-invasive support was initiated. At the same time, LUS review revealed reappearance of A-lines in the L/R1–4 regions and a few scattered B-lines. Enteral feeding and caffeine were introduced to reduce the risk of apnea.

On day 4, the infant's oxygen saturation dropped to 85% and did not improve following tactile stimulation. Arterial blood gas analysis showed: pH 7.25, PCO_2_ 79 mmHg, PO_2_ 55 mmHg, lactate 1.3 mmol/L, BE 4.5 mmol/L, and HCO_3_^−^ 34.6 mmol/L. Ventilation mode was changed to synchronized intermittent mandatory ventilation (SIMV) combined with pressure support ventilation (PSV) and volume guarantee (VG). Chest x-ray indicated possible neonatal pulmonary infection (Figure 2b). LUS findings included scattered B-lines in both lungs, with the L5 zone showing extensive shred signs and atelectasis. A small pleural effusion was also visible between the ribs (Figure 2c). Pulmonary hemorrhage was strongly suspected based on imaging, although clinical signs were absent. Typical ultrasound features of pulmonary hemorrhage include: (1) Fragmentation sign, which is the most frequently observed pattern. (2) Lung consolidation with air bronchograms, variable in size depending on the severity. (3) Pleural effusion, which is present in most neonates with pulmonary hemorrhage. Ventilator parameters were actively adjusted, and plasma transfusion was administered. Soon after, bright red fluid was aspirated via the endotracheal tube, confirming a diagnosis of occult pulmonary hemorrhage. Treatment included hemostatic therapy (plasma and hemocoagulase) and prone positioning (10). After 6 hours, LUS reassessment showed resolution of the fragmentation signs and a decrease in the area of atelectasis (Figure 2d). Blood oxygen levels remained stable, though patient-ventilator asynchrony persisted, and ventilator settings continued to be gradually reduced. The endotracheal tube was removed on day 5, and the infant was discharged after 62 days of hospitalization (July 11, 2024). At follow-up, the child weighed 2,950 g (p8), with stable oxygen saturation and no need for oxygen therapy.

### 3.3 Case 3

A 44-minute-old male infant was admitted with respiratory distress following birth through Grade III meconium-stained amniotic fluid, accompanied by birth asphyxia. During resuscitation, meconium was suctioned from the endotracheal tube before the infant was transferred to the NICU（when applicable）. Umbilical cord blood gas analysis showed a pH of 7.20. The diagnosis on admission was Meconium Aspiration Syndrome (MAS) (October 4, 2024).

#### 3.3.1 Key ultrasound features of MAS include

(1) Widespread, multifocal consolidation, the hallmark of MAS, is the most prominent finding on ultrasound. The consolidation appears unevenly distributed, typically more pronounced in dorsal regions than ventral. The borders of these consolidated zones may be irregular or serrated. As lung injury worsens, the consolidation expands. Larger affected areas often show fragmentation signs and air bronchograms. In severe cases, consolidation may appear as localized atelectasis, with a relatively uniform liver-like echotexture at the edge of the lesion and visible air or fluid bronchograms within. (2) Consolidated areas resulting from inflammation, poor alveolar inflation, and pleural abnormalities often exhibit extensive pleural line changes, disappearance, discontinuity, thickening, or blurring. A-lines vanish beneath and around these regions, replaced by dense B-lines. (3) Most non-consolidated areas present B-lines or signs consistent with alveolar-interstitial syndrome (AIS). In advanced MAS, pulmonary edema becomes more evident, sometimes producing dense B-lines or even a “white lung” pattern. Localized A-lines may also be seen due to patchy lung involvement, which can suggest focal emphysema. (4) In some severe cases of MAS involving intense pulmonary inflammation and exudation, pleural effusion may also be present.

#### 3.3.2 Physical examination

At the time of evaluation, the infant's vital signs were as follows: temperature (T) 36.7°C, pulse (P) 120 beats per minute, respiration (R) 65 breaths per minute, and blood pressure (BP) 45/23 mmHg (mean arterial pressure: 31 mmHg). The infant weighed 3.72 kg and showed features typical of a full-term newborn, with normal responsiveness. Bilateral respiratory excursions were symmetrical, with evident chest retractions. Breath sounds were increased on both sides, accompanied by moist rales. Cardiac auscultation revealed a regular rhythm, strong heart sounds, and no murmurs in any valve area. The abdomen was slightly distended but soft, with no umbilical bleeding. Neurological findings were unremarkable.

#### 3.3.3 Primary treatment course

**Figure 3:**
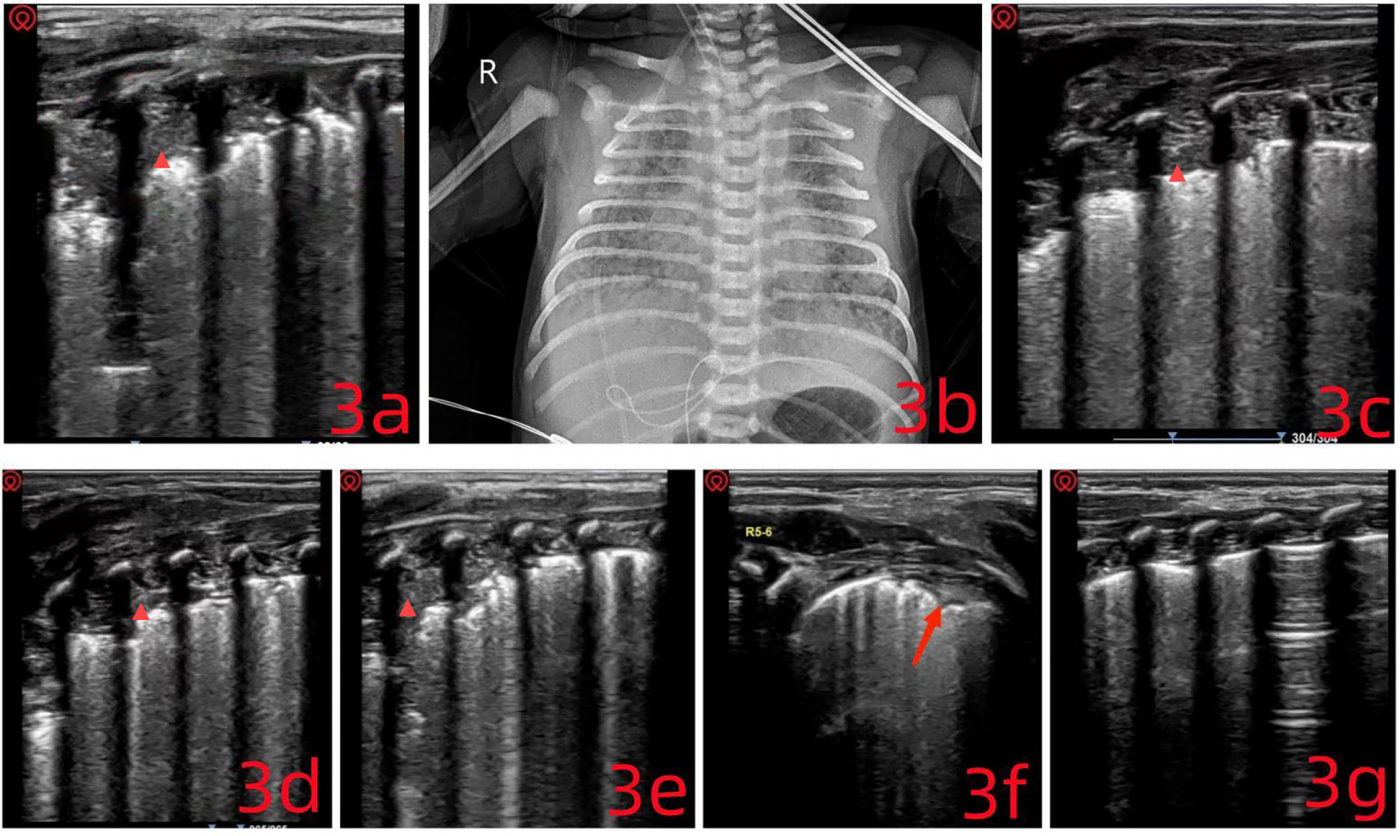
**(a)** multiple pulmonary consolidations of varying sizes are visible (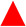), extending across four intercostal spaces, with a maximum depth of 1.2 cm. Given the clinical context, these findings are consistent with consolidations related to meconium aspiration syndrome. **(c–e)** The consolidated areas show progressive reduction. **(f)** Only a small subpleural consolidation remains. **(g)** The lung ultrasound image is nearly normal.

Lung ultrasound (LUS) was performed immediately after admission. The anterior chest regions (L/R1–4) showed absent A-lines and interrupted pleural lines. Fusion B-lines were visible, along with serrated, small consolidations of varying sizes across multiple intercostal spaces, findings often associated with pulmonary hemorrhage, characterized by extensive subpleural fragmentation. Dynamic air bronchograms were observed in the posterior lung fields (L/R5–6). In these areas, the pleural line was absent, and widespread lung consolidation resembling ‘hepatization’ extended across 4–5 rib spaces. These LUS findings were indicative of either meconium aspiration syndrome or atelectasis (Figure 3a). Chest x-ray further supported the suspicion of fetal aspiration (Figure 3b) (11).

Given the extensive pulmonary consolidation and widespread atelectasis observed on LUS, an ultrasound-guided bronchoalveolar lavage was performed without delay: (1) The patient was placed in a supine position under tracheal intubation. (2) A volume of 2–3 ml of 0.9% normal saline was instilled through the endotracheal tube. (3) Ventilator settings were increased for 15–20 minutes. (4) Airway secretions were aspirated under negative pressure.

Following this, high-frequency oscillatory ventilation was initiated upon confirmation of adequate lung inflation. A closed suction catheter was connected, and nebulization therapy was started. LUS was repeated every 0.5–1 hour, and the above steps were carried out repeatedly. After each round of intervention, LUS was used to reassess the lungs (Figures 3c–e). For regions with poor drainage, the patient's position was adjusted based on ultrasound findings to improve clearance. Six hours into treatment, ventilatory stimulation was reduced to avoid triggering persistent pulmonary hypertension of the newborn (PPHN). LUS findings showed a clear reduction in pulmonary consolidation and atelectasis (Figures 3f,g). By day 3, the invasive ventilation tube was replaced with a non-invasive device, and the infant was discharged one week later (October 12, 2024). During follow-up, the infant demonstrated steady weight gain, stable respiratory status, and normal blood oxygen saturation, with no notable respiratory symptoms.

## 4 Discussion

### 4.1 Strategies for rapid identification of atelectasis

Traditional imaging methods such as x-ray lack both the sensitivity and specificity required for reliable detection of atelectasis, often resulting in underdiagnosis. Factors including patient positioning, segmental lung involvement, and limited cooperation in neonates further complicate accurate diagnosis (11, 12). Additionally, x-ray imaging exposes neonates to ionizing radiation. In Case 1 (Figure 1b), atelectasis was initially misinterpreted as thymic tissue. While CT provides greater sensitivity, it carries a higher radiation burden and is impractical for repeated bedside evaluations.

Lung ultrasound (LUS) demonstrates high specificity in diagnosing atelectasis (11), with the appearance of lung tissue “hepatization” serving as a key indicator. LUS combines advantages of both x-ray and CT, allowing for repeated bedside assessments that provide rapid quantification of atelectatic regions (13). It enables real-time mapping of affected lung areas and offers insight into disease progression and prognosis.

## 5 Strategies for guiding pulmonary recruitment

Atelectasis in neonates arises from various underlying conditions. LUS allows for the clear identification of specific respiratory disorders, accelerating the process of identifying the cause and refining treatment strategies. Since the mechanisms behind neonatal respiratory diseases differ, pulmonary recruitment must be tailored accordingly. For instance, neonatal respiratory distress syndrome is characterized by alveolar collapse and surfactant deficiency, and its management relies on surfactant replacement therapy and support for alveolar stability (14). In contrast, atelectasis in meconium aspiration syndrome (MAS) results from airway obstruction by meconium, requiring strategies focused on its clearance. For neonatal pneumonia, where atelectasis is caused by airway narrowing due to edema and inflammatory secretions, treatment should aim at reducing cellular edema and clearing secretions from the respiratory tract (15).

**Figure 4:**
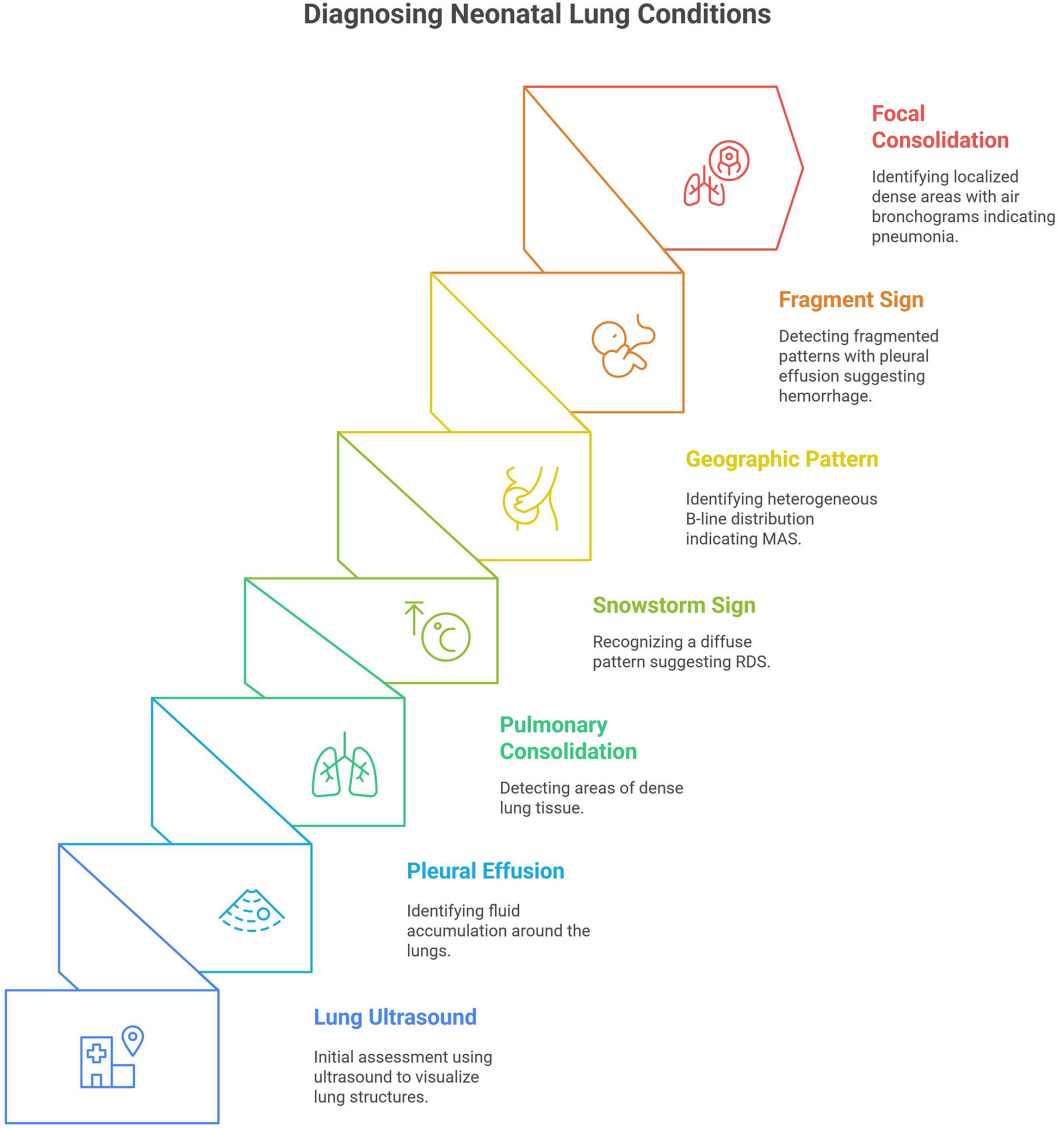
Conceptual framework and diagnostic-therapeutic algorithm for neonatal atelectasis.

Figure 4 outlines an approach for clinicians to quickly differentiate types of atelectasis using LUS. The initial step is identifying whether the affected area presents as a solid or fluid-filled dark region. Based on the features of the consolidation, the underlying condition can be inferred, guiding the development of disease-specific diagnostic and therapeutic plans. In this way, LUS supports precise diagnosis and, when interpreted alongside clinical history, facilitates timely clinical decision-making.

## 6 Comparison with traditional treatment strategies

Neonatal atelectasis often signals a critical condition requiring urgent evaluation (16–18). LUS increases diagnostic accuracy over conventional imaging and supports targeted, effective interventions. Studies have shown that LUS-guided management results in shorter hospital stays and reduced healthcare resource consumption (19, 20).

## 7 Implementation pathway for precision respiratory disease management

The precision approach to managing neonatal atelectasis with the aid of LUS involves a structured operational pathway:

### 7.1 Stratified diagnosis

LUS allows quantification of the extent of atelectasis (e.g., number of involved intercostal spaces) and classification of lesion types, consolidation, exudation, or obstruction. This enables differentiation between infectious causes such as pneumonia and non-infectious ones like meconium aspiration.

### 7.2 Dynamic intervention

Treatment strategies are aligned with the underlying mechanism. Infections may require a combination of anti-inflammatory therapy and airway drainage, while obstructions call for mechanical clearance.

### 7.3 Multimodal integration

LUS findings are combined with blood gas analysis and ventilator waveform data to adjust ventilation settings (e.g., PEEP titration) and guide pharmacologic interventions, such as surfactant or immunoglobulin therapy. This integrated model improves therapeutic efficiency and reduces iatrogenic risks, including repeated intubation (21, 22). Although the COVID-19 case reflects early pandemic data, the approach remains applicable to other emerging pathogens such as RSV and human metapneumovirus, offering a flexible and scalable framework for precision neonatology.

LUS plays a central role in linking imaging features with disease mechanisms to guide individualized management in neonatal atelectasis. For example, in pulmonary hemorrhage, the presence of the “fragment sign”, hypoechoic septations caused by intra-alveolar clots and interstitial edema, reflects the hemorrhage's severity. This indicator supports decisions to reduce mean airway pressure to avoid secondary injury and tailor antifibrinolytic therapy based on coagulation status. In contrast, MAS presents with distinct ultrasound features: multifocal, serrated consolidations and “geographic” distributions of B-lines, representing the combined effects of airway obstruction and alveolar collapse. These spatial patterns inform the application of high-frequency oscillatory ventilation, while dynamic LUS monitoring tracks the formation of “aeration halos” along the consolidation margins following lavage. The low occurrence of pleural effusion in both conditions enhances the diagnostic specificity of LUS. Real-time imaging feedback allows clinicians to make minute-to-minute adjustments, such as modifying bronchial lavage frequency in MAS based on how quickly consolidations recede, or fine-tuning PEEP weaning protocols in pulmonary hemorrhage by monitoring fragment sign resolution. This combined imaging-respiratory mechanics strategy reduces variability in atelectasis resolution time by more than 40% compared with traditional methods and significantly lowers respiratory support needs and complication rates. The approach reflects a successful application of precision medicine principles in neonatal intensive care (Table 1).

**Table 1:** Sonographic features and clinical correlations of neonatal atelectasis by etiology.

Etiology	Ultrasound features	Pathological mechanism	Key references
Pneumonia	- Irregular or blurred pleural line	Inflammatory exudation leading to airway narrowing and alveolar collapse	Liu et al. (2)
	- Focal consolidation with dynamic air bronchograms		
	- Dense B-lines (“white lung sign”)		
Pulmonary hemorrhage	- Shred sign (irregular lung fragmentation)	Vascular rupture with alveolar and interstitial blood accumulation	Liu et al. (10)
	- Subpleural consolidation with pleural effusion		
	- Absence of dynamic air bronchograms		
Meconium aspiration syndrome (MAS)	- Multifocal serrated consolidations	Airway obstruction and chemical pneumonitis caused by meconium	Piastra et al. (11)
	- Heterogeneous B-line distribution (“geographic pattern”)		
	- Rare pleural effusion		
Respiratory distress syndrome (RDS)	- Disappearance of A-lines	Generalized alveolar collapse due to surfactant deficiency	Guo et al. (16)
	- Diffuse “snowstorm sign”		
	- Thickened or blurred pleural line		

## 8 Limitations

Although lung ultrasound (LUS) has shown clear advantages in the management of neonatal atelectasis, several limitations of this study must be noted. First, the diagnostic accuracy of LUS may be affected by image quality, which can vary depending on technical factors such as the infant's positioning, operator experience, and equipment resolution. While a standardized protocol was applied, including the use of the Mindray M9 system and a 12–4 MHz probe, suboptimal image clarity in certain cases may have affected the precise delineation of consolidation margins. Future studies could investigate advanced imaging techniques or post-processing algorithms to improve LUS resolution and reproducibility. Additionally, including its operator dependence, patient positioning, and variability with different levels of ventilatory support need to be considered. There are some treatment regime concerns which is deviation from standard of care. A prospective, multi-center study would be a valuable next step to validate these findings and address some of the limitations inherent in single-center, retrospective case series.

## 9 Conclusion

LUS provides high diagnostic specificity for distinguishing between different causes of neonatal atelectasis based on distinct ultrasound patterns that reflect underlying disease processes. This radiation-free tool supports real-time, bedside evaluation and ongoing monitoring, offering critical guidance for clinical decisions in the management of neonatal respiratory disorders.

## Data Availability

The datasets presented in this study can be found in online repositories. The names of the repository/repositories and accession number(s) can be found in the article/Supplementary Material.
